# Oligodendrocytes and myelination: pioneering new frontiers in cognitive neuroscience

**DOI:** 10.3389/fnins.2025.1618468

**Published:** 2025-07-21

**Authors:** Ning Zhang, Rulan Yi, Fuwang Zhong, Yali Lu, Wenjia Chen, Zhidan Ke, Yi Zhang, Liang Zhou, Pengyu Wang, Wei Li

**Affiliations:** ^1^Department of Anesthesiology, Southwest Hospital, Third Military Medical University (Army Medical University), Chongqing, China; ^2^Key Laboratory of Anesthesia and Organ Protection, Ministry of Education, Zunyi Medical University, Zunyi, China; ^3^Department of Anesthesiology, The Second Affiliated Hospital of Zunyi Medical University, Zunyi, China; ^4^Department of Anesthesiology, The Affiliated Hospital of Zunyi Medical University, Zunyi, China; ^5^Department of Health Statistics, College of Preventive Medicine, Army Medical University, Chongqing, China; ^6^State Key Laboratory of Trauma and Chemical Poisoning, Department of Wound Infection and Drug, Daping Hospital, Army Medical University, Chongqing, China

**Keywords:** oligodendrocytes, myelin, demyelination, remyelination, cognitive function

## Abstract

There has been a growing interest in the role of oligodendrocytes (OLs) and the myelin sheaths they form around axons in cognitive function. Historically, OLs were primarily considered to be involved in axonal insulation and signal transmission within the central nervous system (CNS). However, an increasing body of research indicates that OLs and myelination are integral to neural circuit formation, the regulation of plasticity, and higher-order cognitive functions. Developmental and functional abnormalities in OLs, as well as deficits in myelination, are pathologically associated with diseases characterized by clinical cognitive dysfunction. These abnormalities have significant implications for the development of diagnostic and therapeutic strategies for neurological disorders and for the advancement and innovation of treatment methodologies. Investigations into the impact of OLs and myelination on cognitive function offer a novel perspective for understanding the development, plasticity, and pathophysiological mechanisms of the nervous system. Future research endeavors are anticipated to elucidate the complexities of OLs and myelination, thereby offering renewed prospects for the diagnosis and treatment of neurological disorders. This review provides a systematic examination of contemporary research concerning OLs and myelination, covering fundamental mechanisms, their roles in cognitive function, recent clinical advancements, emerging therapeutic strategies, ongoing scientific debates, key challenges, and future directions. By incorporating multidisciplinary perspectives, this synthesis seeks to establish a comprehensive framework that will guide subsequent investigations in this domain.

## 1 Introduction

The central nervous system (CNS) is composed of complex neural networks, within which glial cell populations function as essential supportive components. These populations are primarily comprised of three distinct cell types: microglia, astrocytes, and oligodendrocytes (OLs; Zhou et al., 2021). Remarkably, glial cells are numerically predominant in the CNS, constituting up to 90% of the total cellular population in neural tissue (Kato et al., 2013). While traditional neuroscience research has predominantly concentrated on neurons and their synaptic plasticity mechanisms, recent evidence indicates that glial cell populations play critical roles in the functional regulation of neural circuits (Suminaite et al., 2019). Notably, OLs have been shown to respond to external signals, including neuronal activity, thereby influencing neurophysiological functions. As the primary effector cells responsible for CNS myelination, OLs exhibit developmental impairments and dysfunctions that are closely linked to the pathogenesis of various neuropsychiatric disorders. With advancements in modern biotechnology, particularly through the innovative application of molecular biology, genetics, and imaging techniques, researchers have gained a deeper understanding of the role of myelination in regulating higher-order neural functions. This article synthesizes recent key research findings to review the regulatory mechanisms of OLs and myelination in cognitive function, as well as their implications in neurological disorders.

## 2 Essential functions of oligodendrocytes and myelination

### 2.1 The basic functions of oligodendrocytes

#### 2.1.1 Oligodendrocytes form myelin sheath

Oligodendrocytes are glial cells in the CNS, a class of multifunctional neuroglia primarily located in the white matter of the CNS (Zhou et al., 2021). As mature, terminally differentiated cells derived from oligodendrocyte precursor cells (OPCs), they extend their cell membranes to wrap around axons, forming multilayered insulating myelin sheaths around neuronal axons (Emery, 2010; López-Muguruza and Matute, 2023). A single oligodendrocyte can simultaneously provide myelin for up to 50 axons (Baumann and Pham-Dinh, 2001), significantly enhancing the efficiency of nerve signal transmission (Yalçın et al., 2024).

#### 2.1.2 Oligodendrocytes provide metabolic support

Oligodendrocytes also deliver metabolic support to axons (Mot et al., 2018; Sock and Wegner, 2021; Tepavčević, 2021). OLs absorb glucose from the bloodstream or adjacent astrocytes via monocarboxylate transporter 1 (MCT1), subsequently converting it into lactate. This lactate is then released into axons through MCT1 to satisfy the neurons’ rappid energy requirements. Research has demonstrated that the knockout of oligodendrocyte-specific MCT1 in mice results in axonal energy deficits and motor impairments (Fünfschilling et al., 2012). Furthermore, OLs are involved in the transport of lipids and cholesterol. Given that myelination necessitates substantial lipid quantities, OLs provide cholesterol to axons by secreting apolipoprotein E (ApoE) and other factors, thereby maintaining the stability of the axonal membrane (Saher et al., 2005).

#### 2.1.3 Oligodendrocytes regulate neural plasticity

Oligodendrocytes play a crucial role in modulating neural plasticity through dynamic signaling interactions, thereby preserving the functional integrity of brain networks and supporting neurodevelopment and cerebral homeostasis (Kato et al., 2013). Empirical evidence indicates that high-frequency neuronal firing, induced by activities such as learning and training, facilitates the differentiation of OPCs into mature OLs, which subsequently form new myelin sheaths around axons. Experimental studies have demonstrated that optogenetic activation of neurons in the mouse prefrontal cortex (PFC) results in increased local myelin thickness (Gibson et al., 2014). Myelinating OLs are capable of monitoring neuronal activity (Micu et al., 2007) and modulating action potential conduction velocity in hippocampal axons of rats (Yamazaki et al., 2007). The process of myelination, which encompasses the proliferation, differentiation, and maturation of OPCs, initiates early in life and continues into adulthood, exhibiting dynamic plasticity. Activity-dependent plasticity in myelinating OLs significantly influences neuronal circuit function (Monje, 2018).

Oligodendrocytes play a pivotal role in enhancing synaptic plasticity through the secretion of neurotrophic factors, such as brain-derived neurotrophic factor (BDNF), and metabolic substrates like lactate, thereby directly influencing neuronal survival and functional activity (Hu et al., 2024; Yalçın et al., 2024). Furthermore, OLs engage in interactions with other glial cells, including astrocytes and microglia, to modulate the CNS microenvironment. Evidence suggests that OLs contribute to the upregulation of blood-brain barrier function, thereby limiting the ingress of potentially harmful substances (Kimura et al., 2020) and maintaining the specialized microenvironment of the CNS. These interactions are crucial for protecting the brain from external insults and preserving its delicate homeostasis. In addition, OLs are integral to CNS repair and regeneration, as they possess the ability to regenerate and replace myelin sheaths around axons following injury—a process known as remyelination. This regenerative capacity is essential for functional recovery after injury and is particularly significant in the context of demyelinating diseases such as multiple sclerosis (MS). The presence of OPCs in the adult CNS is vital for this process, as they can differentiate into mature OLs in response to injury signals (Clayton and Tesar, 2021). Beyond their roles in myelination and repair, OLs are actively involved in glial signaling (Moore et al., 2020; Wellman et al., 2020), which modulates neuronal function (Tanaka et al., 2021; Welsh et al., 2010) and may contribute to the pathogenesis of neurodegenerative and psychiatric disorders (Guest et al., 2015; Raabe et al., 2018). The interaction between OLs and neurons is highly intricate, involving multiple signaling pathways essential for maintaining CNS functionality.

### 2.2 Basic functions of myelin sheath

The myelin sheath is rich in lipids (70%) and proteins (30%), including myelin basic protein (MBP) and proteolipid protein (PLP). These molecules are synthesized by OLs and assembled into tightly compacted lamellar structures (Franklin and Ffrench-Constant, 2008). As a lipid-rich membrane wrapping around nerve fibers, the myelin sheath serves as a critical insulating structure for neuronal axons. Its insulating properties enable saltatory conduction of action potentials between Nodes of Ranvier (Franklin and Ffrench-Constant, 2008; Hartline and Colman, 2007), significantly increasing conduction velocity while reducing energy expenditure and protecting the axons. In addition to supporting fundamental neural processes by enhancing signal velocity, myelin is also pivotal in higher-order cognitive functions, such as learning, memory, and executive control. The lipid bilayer structure of myelin serves to electrically insulate axons from adjacent tissues, thereby preventing signal leakage or cross-talk during impulse conduction and ensuring the fidelity of action potentials. Myelin significantly enhances the velocity of nerve impulse conduction through saltatory conduction (Boda, 2021), a process in which action potentials propagate by leaping between the Nodes of Ranvier. This mechanism eliminates the need for sequential ion channel activation along the axon, allowing myelinated fibers to achieve transmission speeds up to 50 times faster than their unmyelinated counterparts (Paez and Lyons, 2020; Sock and Wegner, 2021; Suminaite et al., 2019; Tiane et al., 2019; Yi et al., 2023). Recent research indicates that OLs can dynamically adjust myelin thickness and internodal length to meet the conduction requirements of specific neural circuits. Although myelination substantially increases the speed of action potential propagation, its precise functional impact is determined by specific structural parameters (Osso and Hughes, 2024). Myelin, the specialized membrane that envelops neuronal axons, is essential for maintaining axonal integrity and functionality (Kent and Miron, 2024). It provides metabolic support to axons and preserves their structural and functional homeostasis (Simons et al., 2024). The enhanced production and transport of lactate by myelin exert neuroprotective effects on axons. The myelin sheaths surrounding these axons form specialized conduits known as “myelin channels,” which facilitate the transport of lactate and other neurotrophic factors to axons, thereby supporting axonal structural maintenance and viability. Oligodendrocytes provide energy substrates, such as lactate, to axons through these myelin channels, meeting their high metabolic demands during neural activity (Ruskamo et al., 2022). Additionally, myelin acts as a physical barrier that shields axons from external neurotoxic substances, such as inflammatory cytokines and oxidative stress byproducts, thereby delaying axonal degeneration. The composition and structural integrity of myelin are crucial for its physiological functions. Myelin basic protein (MBP) plays a vital role in myelin compaction, which is essential for maintaining the insulating properties of myelin sheaths. The interaction between MBP and lipid membranes, particularly in the presence of cholesterol, is critical for the structural stability of myelin. Variations in cholesterol content influence MBP-lipid monolayer interactions, thereby affecting myelin membrane compaction and thermodynamic stability (Träger et al., 2020). Moreover, myelin exhibits heterogeneous distribution and thickness along axons, with significant variations across different neuronal populations. Recent studies have found that OLs in adult CNS can still dynamically adjust myelin thickness and participate in learning and memory. For example, experiments on mice have shown that myelination is associated with complex motor skills (McKenzie et al., 2014).

## 3 Core mechanistic roles of myelination in cognitive function

### 3.1 Structural support and signal conduction efficiency

**FIGURE 1:**
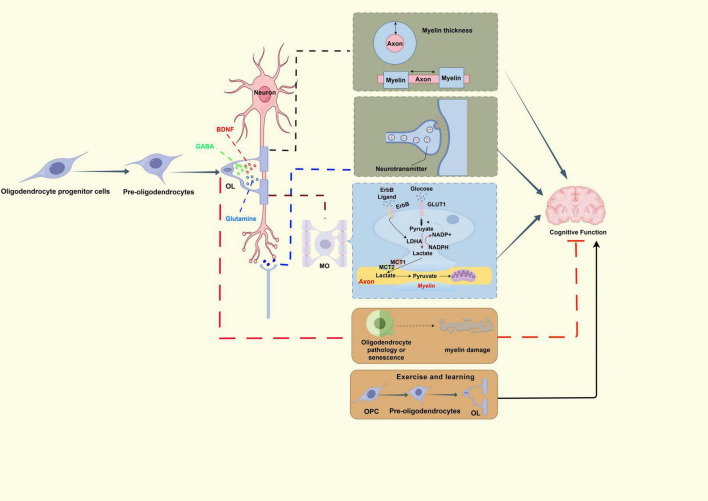
The impact of myelination on cognitive function. (1) Structural support: Oligodendrocytes wrap axons to form myelin sheaths. Myelin sheaths provide insulating structures to protect axons and accelerate neural signal conduction through “skipping conduction.” Changes in axon thickness and myelin sheath spacing affect the speed of neural conduction and have an impact on advanced cognitive functions. (2) Metabolic regulation: Oligodendrocytes provide energy metabolism for axons through aerobic glycolysis; Secondly, the expression of lactate dehydrogenase is regulated through the ErbB signaling pathway, affecting the ability of OLs to transport lactic acid to axons. Meanwhile, neurotransmitters such as glutamate/GABA regulate the differentiation of OPC through receptors and affect myelin, thereby influencing cognitive functions such as learning and memory. The release of neurotrophic factors such as BDNF can regulate the release and metabolism of neurotransmitters, thereby affecting cognitive function. (3) Neural plasticity and developmental regulation: Enhancing cognitive function: exercise and complex learning can induce OPC differentiation and promote myelin formation. Cause cognitive dysfunction: For instance, mutations in the ERBB4/NRG1 signaling pathway lead to abnormal differentiation of oligodendrocytes, affecting white matter integrity and resulting in abnormal cognitive function; The senescence of oligodendrocytes is also associated with cognitive impairment. This is not an exhaustive schematic diagram, but it shows that the factors influencing cognitive function are complex. (Arrows (→) indicate positive regulations, and the symbol (T) represents negative regulations).

Myelination plays a crucial role in significantly enhancing the velocity of action potential propagation along axons (Figure 1). As an effective passive electrical insulator, myelin sheaths substantially increase conduction speed (Yamazaki et al., 2010). The conduction velocity of neurons is influenced by factors such as axonal diameter, myelin thickness, and internodal distance (Steadman et al., 2020). By reducing axonal membrane capacitance and resistance, myelin extends the electrotonic length constant of axons, decreases the likelihood of action potential propagation failure, and markedly enhances the efficiency of neural signal transmission (Nave, 2010). Moreover, myelin improves the fidelity of information transmission through its biophysical properties (Alcami and El Hady, 2019; Cohen et al., 2020) and by facilitating metabolic support from OLs to axons (Moore et al., 2020).

### 3.2 Metabolic support and energetic homeostasis

Aerobic glycolysis in OLs is a crucial energy source for axonal metabolism (López-Muguruza and Matute, 2023) (Figure 1). Research conducted by Hu et al. (2024) has elucidated that the Erythroblastic Leukemia Viral Oncogene Homolog (ErbB) signaling pathway plays a regulatory role in the expression of lactate dehydrogenase. This regulation influences the capacity for lactate shuttling from OLs to axons, which is essential for maintaining axonal conduction. Inhibition of the ErbB pathway results in working memory deficits in mice, indicating that cognitive impairments can occur due to metabolic dysfunction, even when myelin remains structurally intact. Lactate functions as both a critical energetic substrate and a metabolic signaling molecule within the brain and astrocytes, significantly contributing to energy transfer, storage, production, and utilization (Wu et al., 2023). In the context of CNS energy metabolism, monocarboxylates such as lactate and pyruvate are released into the perineuronal microenvironment through glia-specific MCT1 transporters. These substrates are subsequently taken up by neurons via neuron-expressed Monocarboxylate Transporter 2 (MCT2) transporters, thereby providing essential energetic substrates for neural activity. Neurotransmitter signaling and neurotrophic factors exert functional effects on oligodendrocyte lineage cells (Mount and Monje, 2017). Neurotransmitter signaling and neurotrophic factors have significant functional impacts on cells within the oligodendrocyte lineage. Oligodendrocyte precursor cells (OPCs) perceive neuronal activity via glutamate receptors, such as AMPA and NMDA receptors, which subsequently influence myelination and play a role in the regulation of learning and memory (Gallo et al., 1996; Wake et al., 2011). Concurrently, OPCs express GABA receptors, allowing the inhibitory neurotransmitter GABA to modulate their differentiation rate, alter myelination processes, and affect cognitive flexibility (Zonouzi et al., 2015). The release of molecules like glutamine and BDNF plays a crucial role in regulating neurotransmitter release and metabolism, thereby influencing cognitive abilities. Research has demonstrated that the knockout of BDNF in the mouse hippocampus results in reduced myelin thickness and diminished performance in spatial memory tasks (Du et al., 2011).

## 3.3 Neuroplasticity and developmental regulation

Oligodendrocytes and myelin play critical roles in modulating neuronal function and plasticity. The remodeling of myelin can influence neural adaptation by modulating action potential conduction (Osso and Hughes, 2024), while simultaneously altering the energetic support available to neurons. OLs contribute to neuronal health, excitability, and potential information processing by supplying essential metabolites. Myelin serves to insulate axons from extracellular metabolites. Consequently, changes in myelin coverage may impact the availability of local metabolites from both myelin and extracellular sources, thereby affecting the energetic dynamics of axons. Adolescence represents a crucial developmental period for myelination, with abnormalities in this process potentially implicated in the pathogenesis of psychiatric disorders. Research in developmental neuroscience indicates a precise synchronization between the timing of visual cortical myelination and the closure of critical periods for neuroplasticity. The completion of myelination during adolescence may permanently regulate synaptic remodeling capacity in adulthood by stabilizing neural circuit architecture (Xin et al., 2024). Adolescent visual deprivation has been shown to inhibit oligodendrocyte maturation, resulting in abnormally heightened neuronal plasticity in the adult visual cortex, thereby underscoring the significance of myelination in circuit stabilization (Xin et al., 2024). Additionally, investigators from the Chinese Academy of Sciences have identified that the ubiquitin ligase Ring Finger Protein 220 (RNF220) is instrumental in regulating oligodendrocyte differentiation and myelination by stabilizing the protein levels of the transcription factors Oligodendrocyte Transcription Factor 1 (Olig1) and Oligodendrocyte Transcription Factor 2 (Olig2). Mutations within this regulatory system can result in leukodystrophy and cognitive decline. Dynamic myelin remodeling, characterized by alterations in myelin thickness and internodal length, is a fundamental mechanism that facilitates environmental adaptation and learning. Animal studies have demonstrated that the acquisition of motor skills, such as rotarod training in mice, promotes the differentiation of OPCs into mature OLs in specific brain regions, including the motor cortex (Figure 1). This process enhances myelination, thereby increasing conduction velocity and supporting memory consolidation (Bacmeister et al., 2020). Following complex maze tasks, mice exhibit a significant increase in myelination within the corpus callosum, while the blockade of OPC differentiation impairs long-term memory storage (Chen et al., 2019). In neuroscience research, the T1-weighted/T2-weighted (T1w/T2w) ratio is frequently employed to quantify the extent of white matter myelination. An elevated ratio signifies increased myelin density, serving as a biomarker for evaluating neurodevelopmental integrity or demyelinating conditions. This metric is also utilized in the assessment of neurodegenerative diseases, such as Alzheimer’s disease (AD), and psychiatric disorders, offering insights into microstructural neural alterations. Clinical neuroimaging studies utilizing T1w/T2w ratio mapping reveal that non-linear progression of white matter myelination from childhood to adolescence significantly correlates with enhanced executive function and language processing abilities, suggesting that myelination could be a key driver (Dipnall et al., 2024).

## 4 Pathological correlations between myelination defects and cognitive dysfunction

### 4.1 Schizophrenia (SZ)

Schizophrenia is a complex neurodevelopmental disorder involving the interplay of genetic, environmental, and neurobiological factors, characterized by significant disturbances in thinking, perception, emotion, behavior, and social functioning. Recent studies have revealed that oligodendrocyte (OL) dysfunction and myelination abnormalities play a critical role in the pathological mechanisms of schizophrenia, potentially affecting neural signal transmission, synaptic plasticity, and brain network integration.

Oligodendrocyte and myelination abnormalities in schizophrenia may impair cognitive and emotional functions through mechanisms such as white matter connectivity deficits, impaired synaptic plasticity, and neuroinflammation. Research indicates that oligodendrocyte abnormalities in schizophrenia patients may be associated with reduced oligodendrocyte density, such as a significant decrease in OL numbers in key brain regions like the PFC, hippocampus, and corpus callosum, along with downregulated OLIG2 expression. Concurrently, schizophrenia exhibits abnormal expression of myelin-related genes, with microarray analyses showing reduced expression of genes such as myelin basic protein (MBP) and PLP in the brains of schizophrenia patients (Tkachev et al., 2003), as well as decreased levels of CNP (2’,3’-cyclic nucleotide 3’-phosphodiesterase, an OL marker) protein (Flynn et al., 2003).

Neuroimaging studies suggest that white matter abnormalities in schizophrenia patients are closely linked to impaired oligodendrocyte function. Research has demonstrated that the ERBB4/NRG1 signaling pathway is frequently mutated in schizophrenia patients, affecting oligodendrocyte differentiation and thereby compromising white matter integrity, ultimately leading to cognitive dysfunction (Hu et al., 2024). Studies have found that disruption of ErbB signaling impairs oligodendrocyte myelination and aerobic glycolysis, resulting in working memory deficits. The loss of ErbB signaling in OLs reduces axonal myelin sheath thickness and slows conduction velocity (Dong and Zhen, 2015). Specific inhibition of the ErbB signaling pathway in OPCs leads to structural defects in myelin, directly associated with working memory impairment (Hu et al., 2024). When ErbB signaling is blocked in OLs *in vivo*, changes in oligodendrocyte quantity and morphology are accompanied by reduced myelin thickness, slower CNS axonal conduction velocity, and behavioral alterations (Roy et al., 2007).

Mechanistically, ErbB inhibition attenuates K-Ras activity, leading to decreased expression of lactate dehydrogenase A (LDHA), which is essential for promoting aerobic glycolysis in mature OLs. Supplementation with L-lactate can restore axonal conduction and working memory capacity impaired by ErbB inhibition in mature OLs (Hu et al., 2024). These findings suggest that abnormal myelination may contribute to the cognitive dysfunction observed in schizophrenia. Furthermore, oligodendrocyte dysfunction has been implicated in both schizophrenia and depression. Research indicates that antipsychotic medications, such as haloperidol, olanzapine, and quetiapine can promote the differentiation of OPCs into mature OLs by modulating transcription factors Olig1 and Olig2 (Fang et al., 2013). This process promotes oligodendrocyte development, improves myelin integrity (Zhang et al., 2012), and alleviates cognitive dysfunction (Gingele and Stangel, 2020).

### 4.2 Alzheimer’s disease (AD)

In Alzheimer’s disease, oligodendrocyte function is compromised by various factors, including aging, aluminum toxicity, small vessel disease with associated white matter injury, and amyloid-beta (Aβ) pathology. Importantly, alterations in myelination patterns and oligodendrocyte function are evident in the early stages of AD, preceding the formation of detectable Aβ plaques and tau pathology. Research indicates a significant reduction in myelin density within brain regions affected by AD, such as the hippocampus and anterior cingulate cortex, with the degree of myelination loss positively correlating with the severity of cognitive impairment. Myelin pathology may exacerbate neurodegenerative processes by disrupting axonal metabolic support and compromising neural circuit stability. Oligodendrocyte dysfunction becomes apparent in the early stages of AD, primarily manifesting as myelin loss and dysregulation of lipid metabolism. Notably, simultaneous processes of myelination breakdown and repair occur throughout the progression of AD. Studies conducted on AD model mice demonstrate that although there is an accelerated compensatory myelination response, it remains insufficient to counteract myelin degradation, suggesting that cognitive decline is associated with the dynamics of myelin turnover (Chen et al., 2021). Cutting-edge single-nucleus RNA sequencing of postmortem brain tissue identifies IL-12 signaling as a driver of AD pathogenesis, unveiling potential therapeutic targets.

### 4.3 Multiple sclerosis (MS)

Multiple sclerosis constitutes a distinct category of CNS disorders, characterized by progressive myelin degradation and persistent neuroinflammatory responses. The process of demyelination in MS is directly linked to cognitive decline. Research indicates that the primary impediment to remyelination in chronic MS lesions is impaired oligodendrocyte maturation. The terminal differentiation of OLs is meticulously regulated by both cell-intrinsic mechanisms and microenvironmental cues. Dysfunction in the differentiation of OPCs undermines remyelination capacity, resulting in axonal damage and neuronal loss, which ultimately leads to irreversible neurological deficits—a hallmark of progressive MS. Meanwhile, studies have also found that alterations in glucose metabolism affect the energy supply required for oligodendrocyte function and myelin synthesis (López-Muguruza and Matute, 2023). During development, thyroid hormone (TH) promotes myelination by enhancing oligodendrocyte differentiation. Both TH and sobetirome stimulate remyelination in demyelination models, and long-term treatment significantly improves remyelination repair and motor recovery (Hartley et al., 2019).

## 5 Therapeutic strategies and future directions

### 5.1 Physical therapy for promoting myelin remodeling

**FIGURE 2:**
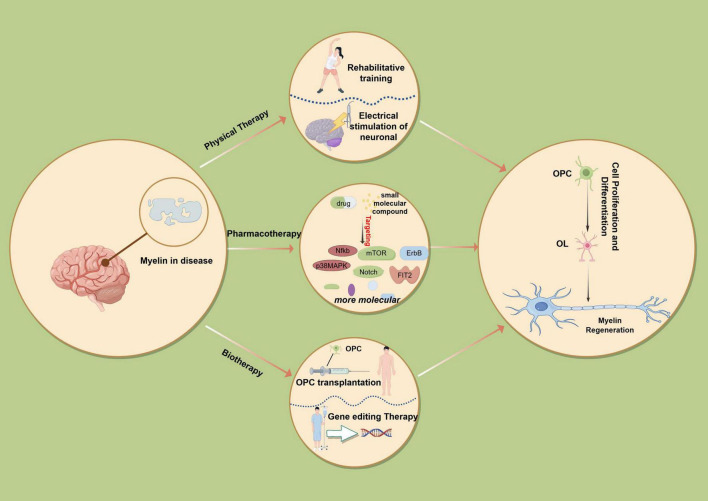
Therapeutic strategies and future directions. (1) Physical therapy aimed at enhancing myelin remodeling involves various neuronal activities, such as motor learning and exercise, which can facilitate the proliferation and differentiation of oligodendrocyte precursor cells (OPCs) and modulate the distribution of newly formed myelin. Additionally, electrical stimulation of neuronal activity has been shown to promote OPC proliferation and myelin formation *in vitro*. Furthermore, targeted manipulation of specific signaling pathways, including the inhibition of NFκB, p38 MAPK, Notch, and BCR pathways, as well as mitochondrial respiration or ATP receptors, and the activation of ErbB and PI3K-AKT-mTOR pathways, alongside targeting the FIT2 protein and autophagy signaling pathways, can enhance cognitive function. Gene and cell therapy approaches, such as the modification of *MED23* or *Sp1* genes, *RNF220* gene editing, and the transplantation of OPCs or NSCs, also hold promise for improving cognitive function.

Myelin repair can be facilitated by modulating the differentiation of OPCs or by enhancing the metabolic functions of OLs. Research indicates that neuronal activity can stimulate the proliferation and differentiation of OPCs, as well as influence the spatial distribution of newly formed myelin sheaths. Animal studies have demonstrated that exercise training can increase the turnover rate of OLs during remyelination and improve the efficiency of myelin regeneration (Bacmeister et al., 2020). Clinical investigations utilizing magnetic resonance imaging (MRI) have revealed that structural changes in white matter, including myelin production or remodeling, are linked to specific types of learning, particularly motor skills such as playing musical instruments or juggling (Bergles and Richardson, 2015). Furthermore, *in vitro* studies have shown that electrical stimulation of neuronal activity can enhance the proliferation and myelin formation of OPCs (Ronzano et al., 2020) (Figure 2).

### 5.2 Targeting signaling pathways

#### 5.2.1 Inhibition of NF-κB and p38 MAPK pathways

Research indicates that targeting the Nuclear Factor Kappa-Light-Chain-Enhancer of Activated B Cells (NF-κB) and p38 Mitogen-Activated Protein Kinase (p38 MAPK) signaling pathways significantly diminishes the release of pro-inflammatory cytokines IL-1β and TNF-α. Andrographolide (AGP), an active compound derived from Andrographis paniculata, inhibits the NF-κB and MAPK pathways, thereby reducing the production of inflammatory mediators, alleviating neuroinflammation, and enhancing cognitive function (Cai et al., 2022). Similar mechanisms have been validated in MS models, where the inhibition of these pathways reduces neuroinflammatory damage to OLs (Zaghloul et al., 2020).

#### 5.2.2 Modulation of ErbB and PI3K-AKT-mTOR signaling pathways

Currently, research on the ErbB and NRG1 signaling pathways has identified them as promising targets for intervention. The work of Tao Yanmei’s team indicates that modulation of the ErbB pathway may enhance cognitive function by facilitating both structural repair and metabolic support (Gibson et al., 2014). Neurotransmitters, including ATP and glutamate, along with growth factors such as Neuregulin-1, influence the bioenergetic metabolism and myelin-generating capacity of OLs through the activation of pathways like Phosphatidylinositol 3-Kinase - Protein Kinase B - Mechanistic Target of Rapamycin (PI3K-AKT-mTOR). These pathways play a critical role in maintaining myelin sheath thickness stability and adapting internodal length. In animal models, targeting the Fat Storage-Inducing Transmembrane Protein 2 (FIT2) protein to reduce lipid droplet accumulation in microglia has been shown to indirectly enhance oligodendrocyte function and mitigate cognitive decline. Furthermore, small molecule drugs, such as clemastine (Li et al., 2021), have been found to inhibit mTOR signaling, thereby promoting the differentiation of OPCs into mature OLs and improving cognitive performance. The elucidation of cellular and molecular mechanisms governing OPC differentiation and myelin repair and regeneration offers novel potential intervention targets for the treatment of MS.

#### 5.2.3 Targeting autophagy signaling pathways

Recent research led by Professor Quanhong Ma at Soochow University has uncovered that oligodendrocyte lineage cells sustain their population and myelin integrity through the process of autophagy. Autophagic activity within these cells is critical for regulating oligodendrocyte (OL) numbers and ensuring myelin stability throughout both brain development and aging. During early developmental stages, autophagy within the oligodendrocyte lineage prevents the overproduction of OLs, whereas in aging, it maintains myelin integrity by facilitating the turnover of myelin structural proteins (Chen et al., 2025). Targeting genes related to autophagy has the potential to delay myelin degeneration and promote regeneration (Aber et al., 2022). These findings highlight complex therapeutic targets for the maintenance of myelin homeostasis.

#### 5.2.4 Targeting the Wnt/β-catenin signaling pathway

Lysine (K)-Specific Demethylase 5C (KDM5C) plays a crucial role in ensuring the timely differentiation of neural progenitors into intermediate precursors by modulating Wingless-related integration site (Wnt) signaling, thereby preserving the neurogenic rhythm. Mutations in KDM5C disrupt the homeostasis of the Wnt signaling pathway, leading to intellectual disabilities. However, pharmacological inhibition of the Wnt pathway, such as with CHIR99021, has been shown to restore cognitive function in mouse models (Karwacki-Neisius et al., 2024). Targeting the downstream effector Transcription Factor 7-Like 2 (TCF7L2) within the Wnt signaling cascade enhances oligodendrocyte differentiation, presenting a novel therapeutic approach for remyelination in MS. Research indicates that TCF7L2 promotes oligodendrocyte maturation by inhibiting autocrine Bone Morphogenetic Protein 4-mediated (BMP4-mediated) signaling (Zhang et al., 2021), suggesting that modulation of this pathway could potentially overcome differentiation arrest in MS lesions.

#### 5.2.5 Inhibiting the notch signaling pathway

Studies have shown that in demyelinated lesions of MS patients, oligodendrocyte progenitor cells proliferate but do not adequately remyelinate. Investigations using cuprizone-induced murine models of demyelination have highlighted the critical roles of various signaling pathways, such as Notch, in oligodendrocyte differentiation and myelin repair. Activation of the Notch pathway inhibits the differentiation of Neuron-Glial Antigen 2 (NG2 +) cells, thereby impairing remyelination (Li et al., 2022; Wang et al., 2017). Conversely, suppression of Notch1 in OLs increases the number of mature OLs in the developing CNS gray matter (Givogri et al., 2002). Interestingly, following CNS injury, NG2 + cells exhibit neurogenic potential, suggesting a dual-repair strategy (Boulanger and Messier, 2017). Nevertheless, Notch signaling is known to negatively regulate neuronal differentiation. Its inhibition promotes neuronal maturation and functional recovery (Chen et al., 2015).

#### 5.2.6 Targeted inhibition of BCR signaling, mitochondrial respiration, or ATP receptors

Pathogenic Granulocyte-Macrophage Colony-Stimulating Factor-Positive B Cells (GM-CSF + B cells) demonstrate increased sensitivity to the inhibition of mitochondrial respiration compared to protective B cell subsets. Inhibition of mitochondrial respiration reestablishes the balance of B cell populations in patients with MS while reducing their pathogenic functions. Attenuation of B cell mitochondrial respiration significantly mitigates disease progression in murine models of neuroinflammation. Targeted inhibition of B cell receptor (BCR) signaling, mitochondrial respiration, or ATP receptors may offer therapeutic benefits for MS (Li et al., 2024) (Figure 2).

### 5.3 Gene and cell therapies

#### 5.3.1 Gene editing therapeutics

Recent clinical investigations have identified that point mutations at various sites within the human Mediator Complex Subunit 23 (*Med23*) subunit are strongly associated with neurodevelopmental and functional abnormalities. Utilizing gene-editing technologies, researchers have developed a *Med23* mutant mouse model, which demonstrates that *Med23* mutations lead to characteristic hypomyelination phenotypes, including cerebral white matter atrophy and cognitive decline. Both *MED23* mutations and deletions hinder the differentiation of OPCs into mature OLs, thereby disrupting myelin formation and regeneration. The integration of gene-editing technology with *MED23* or Specificity Protein 1 *(Sp1)* genetic modifications in stem cells significantly enhances their differentiation into functional OLs, thereby improving the efficiency of remyelination. The *MED23* gene facilitates the expression of genes related to oligodendrocyte differentiation by modulating the activity of the Sp1/P300 complex, which in turn promotes myelination. Animal studies have shown that mice with *Med23* mutations exhibit hypomyelination and cognitive impairments; however, the functional restoration of *MED23* through gene editing has been observed to restore oligodendrocyte maturation and myelin regeneration (Zhang et al., 2024). Myelin repair may be facilitated through the regulation of oligodendrocyte precursor cell (OPC) differentiation or by enhancing the metabolic functions of OLs. Recent studies have identified microRNA (miRNA; Ngo and Kothary, 2022) as a critical regulatory factor in oligodendrocyte development, affecting cell size, proliferation, differentiation, and myelin formation. miRNA-based therapies (Yavarpour-Bali et al., 2020) have shown promise in promoting the differentiation of OPCs into mature OLs and enhancing cognitive function (Huang et al., 2024; Zaghloul et al., 2020). Additionally, RNF220 has been identified as a novel E3 ubiquitin ligase for α-Amino-3-hydroxy-5-methyl-4-isoxazolepropionic Acid (AMPA) receptors, which is integral to excitatory synaptic transmission, synaptic plasticity regulation, and learning and memory functions (Ma et al., 2022). Homozygous mutations in *RNF220* (*R363Q* and *R365Q*) have been associated with abnormal brain function (Sferra et al., 2021). Neurobehavioral analyses indicate altered learning and memory capabilities in *RNF220*-deficient mice, with mutations in the *RNF220* gene contributing to neurodevelopmental disorders such as intellectual disability. The application of gene editing technology targeting *RNF220* holds significant potential for advancing neural function improvements in the future (Figure 2).

#### 5.3.2 Cell transplantation therapy

Oligodendrocyte precursor cell (OPC) transplantation shows promise for remyelination in animal models, as OPC grafts during post-ischemic recovery enhance endogenous oligodendrogenesis and synaptic remodeling through Netrin-1 secretion, resulting in long-term neurological improvements (Yang et al., 2023). *In vitro*-differentiated OPCs transplanted into lesioned areas directly enhance remyelination, effectively repairing demyelinated lesions after spinal cord injury. Neural stem cells (NSCs), capable of differentiating into glial cells, neurons, and astrocytes, are located in the hippocampal dentate gyrus, lateral ventricles, and spinal cord’s central canal. NSCs secrete neurotrophic factors that promote cell survival and support myelin regeneration by enhancing oligodendrocyte differentiation (Albu et al., 2021) (Figure 2).

## 6 Discussion

The functions of OLs are notably diverse, presenting considerable challenges to research in this area. The impact of OLs on cognitive function is multifaceted. From the perspective of neural signal transmission, the extent of oligodendrocyte-mediated myelination is closely linked to cognitive function. Myelination significantly enhances the conduction velocity of action potentials along axons by serving as a passive electrical insulator (Yamazaki et al., 2010). Moreover, ion channels and neurotransmitter receptors expressed on OLs play active functional roles in activity-dependent potentiation of neural signal transmission. Experimental evidence demonstrates that OLs can dynamically monitor neuronal activity and facilitate axonal conduction through non-myelinating mechanisms (Yamazaki et al., 2010). This reveals a sophisticated synergy between myelination and intrinsic oligodendrocyte functions in regulating neural circuit activity, which ultimately manifests as measurable modifications in cognitive performance. Neuroimaging studies of cognitively intact middle-aged adults with familial dementia risk reveal that the estimated years to probable dementia onset correlate significantly with white matter microstructural abnormalities. These pathological changes were characterized by reduced fractional anisotropy and increased mean/radial diffusivity - particularly prominent in the corpus callosum - suggesting a direct correlation between oligodendrocyte/myelin-related white matter alterations and cognitive functional changes (Mak et al., 2021). Notably, in disease models such as APP/PS1 transgenic mice, anti-LINGO-1 antibody treatment has been shown to promote oligodendrocyte maturation and enhance myelin density in the hippocampal formation, concomitant with significant cognitive improvement. These findings provide compelling evidence for the crucial role of OLs and myelination in cognitive function (Yang et al., 2022).

In terms of cellular interactions, disrupted communication between OLs and other cell types can impair cognition. For example, in chronic cerebral hypoperfusion models, inhibited differentiation of OPCs, white matter damage, and cognitive deficits were all linked to impaired oligodendrocyte-astrocyte interactions mediated by BDNF, highlighting the importance of normal intercellular communication between OLs and other cells for cognitive function (Magami et al., 2019). From a metabolic regulation perspective, certain transcriptional regulators critically influence oligodendrocyte function and cognition. For instance, the zinc finger protein ZFP488, an oligodendrocyte-enriched transcriptional regulator, promotes oligodendrocyte differentiation in the developing neural tube and oligodendrocyte lineage (Cui et al., 2024). Its deficiency leads to delayed CNS myelination during development and impaired remyelination after injury (Soundarapandian et al., 2011), ultimately affecting cognition, as precise myelination timing is essential for efficient neural communication and cognitive development (Cui et al., 2024). Furthermore, abnormal expression of certain proteins in OLs can also impair cognition. For example, studies on amyotrophic lateral sclerosis (ALS) have demonstrated that overexpression of mutant TDP-43 in OLs leads to oligodendrocyte damage and motor dysfunction, accompanied by downregulation of cognition-related oligodendrocyte marker genes and cholesterol-associated genes. This indicates that protein dysregulation within OLs may also contribute to cognitive deficits (Horiuchi et al., 2024). From a neurodevelopmental perspective, the dynamic progression of OLs and myelination also significantly influences cognitive function. Studies have revealed that during neural development, the precise timing of oligodendrocyte maturation and myelination is critical for cognitive development. Conversely, during aging, the capacity of OLs in the aging brain to generate and maintain healthy myelin declines (Sams, 2021). The functional deterioration of OLs and myelin impairment are closely associated with the onset of cognitive deficits. Further investigation into the molecular mechanisms and regulatory factors underlying these dynamic changes may provide novel strategies for preventing and treating age-related cognitive disorders.

Certainly, the heterogeneity of OLs further complicates research efforts. Oligodendrocytes exhibit distinct gene expression profiles and functional properties depending on their developmental stage and brain region. For instance, in the embryonic mouse forebrain, oligodendrocyte progenitor cells of different developmental origins and their progeny demonstrate significant functional divergence, making it challenging to generalize findings regarding their roles (Foerster et al., 2024). Moreover, investigating the intricate interactions between OLs and other cell types presents additional challenges. Oligodendrocytes engage in extensive communication and interaction with neurons, astrocytes, and microglia. These interactions exhibit dynamic changes under various physiological and pathological conditions, rendering precise analysis challenging. For instance, in the context of neurodegenerative diseases, the manner in which interactions among multiple cell types collectively influence oligodendrocyte function and disease progression remains inadequately understood (Chu et al., 2021). Several critical points of contention exist within the myelination process. In terms of the regulatory mechanisms governing myelination, although numerous signaling pathways and molecules have been identified as contributors, the specific regulatory details and interactions remain subjects of debate. For instance, the role of Fibroblast Growth Factor-2 (FGF2) in myelination remains controversial. Some studies demonstrate that in chronic demyelination models, FGF2-deficient mice exhibit reduced axonal atrophy, suggesting that attenuated FGF2 signaling promotes neuroprotection and remyelination (Armstrong et al., 2006; Larson et al., 2018; Tobin et al., 2011). Conversely, elevated FGF2 levels induce severe disruption of mature OLs and significant myelin loss (Larson et al., 2018). Clinical observations further complicate this picture: while serum FGF2 levels are elevated in MS patients, its precise function in myelination—whether promotive or inhibitory—remains unclear. These conflicting conclusions across studies may stem from variations in experimental models, target populations, and other methodological factors (Jari et al., 2022).

Future research in the field of OLs and myelination presents numerous potential breakthrough opportunities. Regarding disease treatment targets, comprehensive investigations into the signaling pathways associated with OLs and myelination are anticipated to uncover additional therapeutic targets. A promising avenue for advancement involves elucidating methods to direct transplanted cells or endogenous oligodendrocyte progenitor cells to accurately migrate to sites of injury and differentiate effectively into mature OLs, thereby facilitating efficient myelin regeneration. Additionally, research focused on modulating the microenvironment to enhance the survival, proliferation, and differentiation of OLs may yield novel strategies for improving disease prognosis. For instance, by regulating cytokines and immune cells within the inflammatory microenvironment, conditions conducive to oligodendrocyte repair and myelin regeneration can be established (Raffaele et al., 2021).

## 7 Conclusion and outlook

Oligodendrocytes and the process of myelination serve not only as the structural foundation for neural signal conduction but also as a dynamic center for cognitive regulation. This involves coordinated molecular signaling, metabolic support, and regenerative capacity. The integrity of this process is essential for normal neurotransmission and presents viable therapeutic targets for demyelinating disorders. Future research endeavors should incorporate multi-omics data to unravel the spatiotemporally specific mechanisms of myelination within distinct brain regions, such as the PFC, while advancing precision therapies that target the oligodendrocyte microenvironment. Although both pharmacological and cellular interventions demonstrate promising therapeutic potential, further optimization is necessary for successful clinical translation. The integration of cross-disciplinary technologies, such as organoid models and *in vivo* imaging, will facilitate the transition from mechanistic discoveries to clinical applications. These findings shed light on the intricate regulatory networks underlying neural plasticity, thereby establishing a foundation for the optimization of functional neural circuitry.
